# Neuropsin in mental health

**DOI:** 10.1186/s12576-020-00753-2

**Published:** 2020-05-15

**Authors:** Lina Bukowski, Ana M. F. Chernomorchenko, Anna Starnawska, Ole Mors, Nicklas H. Staunstrup, Anders D. Børglum, Per Qvist

**Affiliations:** 1 IPSYCH, The Lundbeck Foundation Initiative for Integrative Psychiatric Research, Aarhus, Denmark; 2 Department of Biomedicine, Aarhus University, Høegh-Guldbergs Gade 10, Aarhus, Denmark; 3 Center for Genomics and Personalized Medicine, Aarhus University, Aarhus, Denmark; 4 Psychosis Research Unit, Aarhus University Hospital, Aarhus, Denmark; 5 Department of Clinical Medicine, Aarhus University, Aarhus, Denmark

**Keywords:** Neuropsin, *KLK8*, Mental disorders, Mental health, Biomarker, Human, Depression, Extracellular matrix, LTP, Gene expression

## Abstract

Neuropsin is a brain-expressed extracellular matrix serine protease that governs synaptic plasticity through activity-induced proteolytic cleavage of synaptic proteins. Its substrates comprise several molecules central to structural synaptic plasticity, and studies in rodents have documented its role in cognition and the behavioral and neurobiological response to stress. Intriguingly, differential usage of *KLK8* (neuropsin gene) splice forms in the fetal and adult brain has only been reported in humans, suggesting that neuropsin may serve a specialized role in human neurodevelopment. Through systematic interrogation of large-scale genetic data, we review *KLK8* regulation in the context of mental health and provide a summary of clinical and preclinical evidence supporting a role for neuropsin in the pathogenesis of mental illness.

## Introduction

The extracellular matrix (ECM) serine protease neuropsin (also known as NP, PRSS19, BSP1 or TADG14), was named after its apparent neuronal expression and its sequence homology to trypsin [1]. Neuropsin possesses the complete triplet (His-Asp-Ser) of the serine protease domain and exhibits proteolytic activity with a trypsin-like substrate specificity [2]. Identified neuropsin substrates comprise: vitronectin [3]; fibronectin [2]; the cell adhesion molecule L1 (L1CAM) [4]; neuregulin-1 (NRG1) [3]; and the Eph receptor B2 (EphB2) [5], all of which localize to the synapse [6]. Accordingly, and like reported for other trypsin-like serine proteases [7, 8], accumulating evidence support a central role for neuropsin in peri-synaptic proteolysis and structural synaptic plasticity [9]. As several of its proteolytic targets have been linked to neurodevelopmental and mental disorders [10, 11], it is thus conceivable that neuropsin governs psychiatry-related synaptic signaling. Here, we review data on regulation of the neuropsin gene *KLK8* in the context of mental health and provide a summary of clinical and preclinical evidence supporting a role for neuropsin in the pathogenesis of mental illness.

## *Klk8* regulation in the brain

**Fig. 1:**
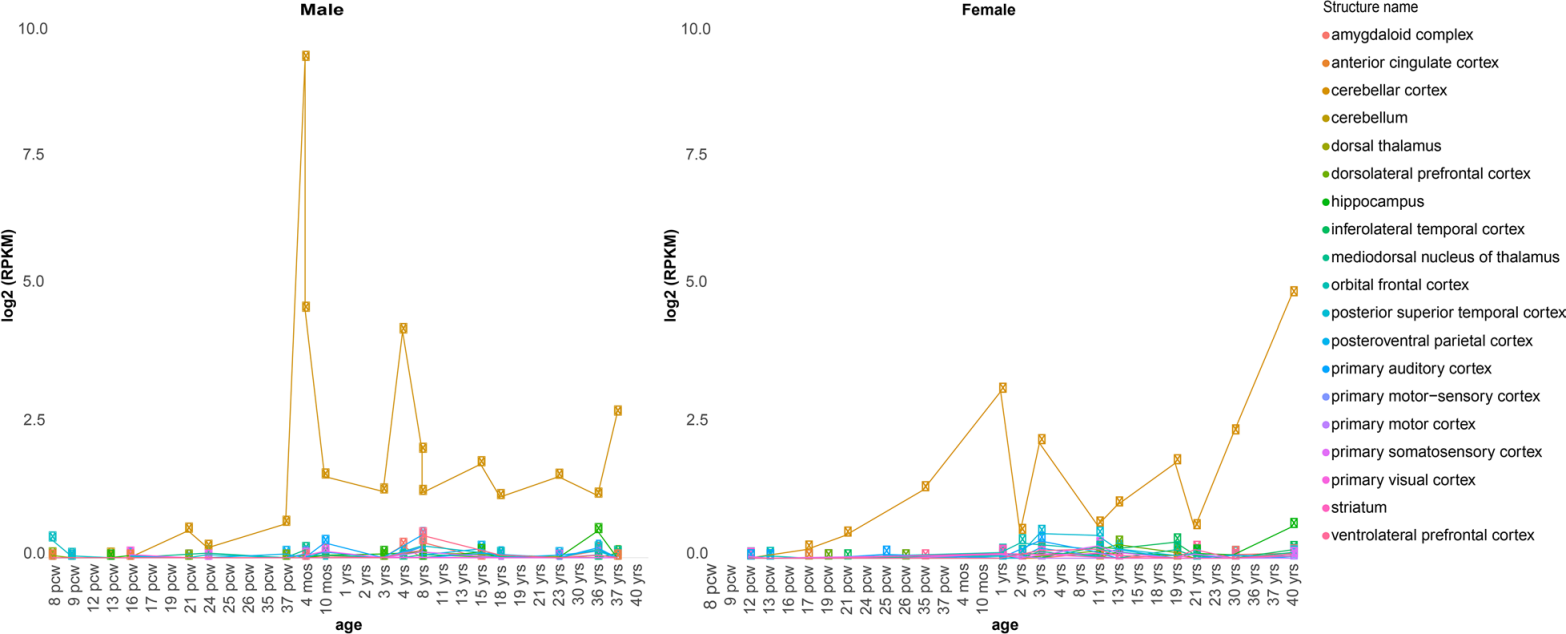
*KLK8* expression across lifespan in the human brain. Shown is the normalized expression of *KLK8* in various human brain tissues across lifespan (from postconceptional week (pcw) 8 to year 40 for females and year 37 for males). Data obtained from [17]

Human *KLK8* mRNA is detected in numerous brain tissues under non-pathological conditions [12] (Fig. 1). Its expression is abundant in the cerebellum throughout life, whereas it peaks postnatally in cortical and limbic tissues, namely in childhood and adulthood [12] (Fig. 1). Age-dependent regional variation in *Klk8* expression and translation has additionally been reported in mice. *Klk8* mRNA as well as protein levels were shown to decrease in the cerebral cortex with age, while peaking in adults in the olfactory bulb and the hippocampus [13]. *Klk8* is susceptible to transcriptional regulation by steroids, as demonstrated by corticosterone in primary cultured hippocampal neurons and in vivo [14, 15], and by estradiol in neuronal and microglial cells [16]. Potentially related to this regulation, a sex bias in hippocampal *KLK8* expression has been reported at the protein level, with higher expression in healthy adult woman than in men [16]. This, however, is not mirrored at the mRNA level in the Brainspan atlas of the developing human brain [17] (Fig. 1) or in adult hippocampal tissue from healthy donors [18].

The *KLK8* transcription start site (TSS) is species and tissue specific [19, 20]. In the developing mouse cortex and midbrain, the primary sources of *Klk8* mRNA are endothelial cells and microglia [21, 22], whereas its expression has been reported in pyramidal neurons of the hippocampal CA1–3 subfields and magnocellular neurons of the lateral/basolateral amygdaloid nucleus on the basis of in situ hybridization immunohistochemistry [1]. Its expression is regulated by neuronal activity [1, 23] and also induced in oligodendrocytes of the spinal cord following injury [24]. A re-analysis of data from the most comprehensive human brain single-cell studies, representing several brain tissues and developmental stages, indicate that *KLK8* is predominantly expressed in neuronal cells, but generally only in a small fraction of cells (Additional file 1: Table S1). Notably, *KLK8* expression was seen in cells derived from temporal cortical tissue in several studies [22, 25], whereas it was not detected in hippocampus-derived cells [18].

**Fig. 2:**
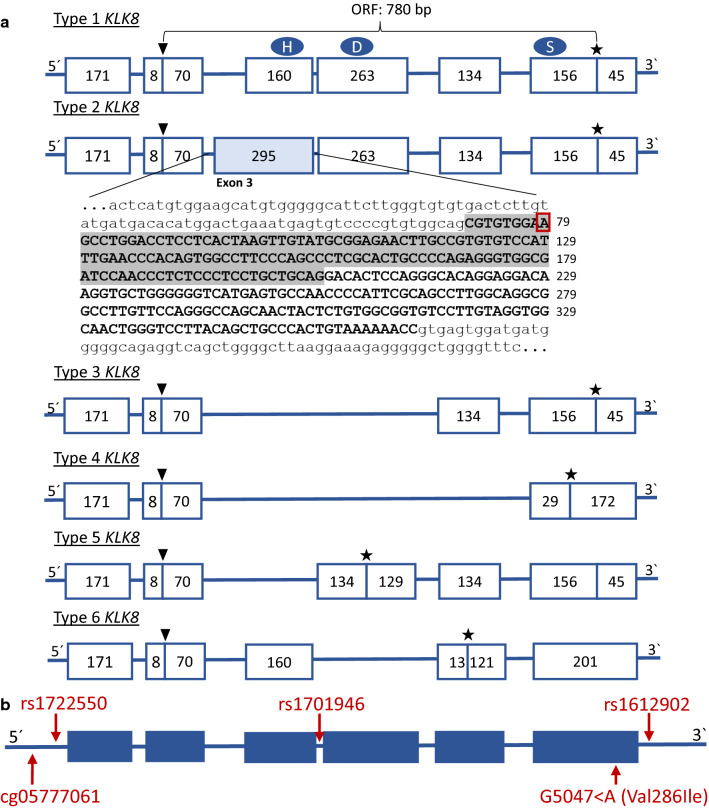
Genomic structures of human *KLK8* transcripts and loci reported in genetic association studies of mental disorders. **a***KLK8* spans 5.4 kb and is located at the long arm of chromosome 19 in human (19q13.4) and comprised 6 exons of which the first is non-coding. *KLK8* cDNA contains a single open reading frame of 780 bp, resulting in a protein comprising 260 amino acids. Shown are exons (boxes) and their length in base pairs, approximate amino acid locations of the characteristic catalytic triad of serine proteases (H, D, and S) as well as start codon (arrow) and stop codon (star). The base pair sequence of exon 3 is shown in upper-case letters (numbers indicate position in coding sequence) and flanking intron sequence in lower-case letters. The base pair sequence specific for type 2 *KLK8* is highlighted in grey and contains the 79 T > A variant (red box). **b** Depicted are identified *KLK8* single nucleotide polymorphisms associated with bipolar disorder (BD), *KLK8* missense mutation identified in schizophrenia, as well as *KLK8* CpG site linked to depression symptomatology in the general population (red arrows)

While orthologs of *KLK8* are found in many species, *KLK8* isoforms (type 2–6, Fig. 2a), expressed due to alternative splicing, have only been reported in humans [26–29]. Among these, both type 1 and 2 are abundantly expressed in the central nervous system (CNS) [26].

Although type 1 and type 2 *KLK8* share the same TSS and lack a splice-form-specific promoter, location of expression appear to be isoform specific. Whereas type 1 is predominantly expressed in the fetal human brain [26], type 2 is detected in embryonic stem cells and embryo brain samples [20], but preferentially expressed in adult brain and particularly in the amygdala and the CA1–3 regions of the hippocampus [26]. Thus, expression of type 2 neuropsin seems to be developmental-stage related. Type 2 *KLK8* results from an in frame splice site mutation in exon 3 that was fixed during human evolution [19, 30]. Specifically, a single T-to-A substitution at position 79 in the coding region (Fig. 2a) leads to a novel GAA-containing motif in exon 3 of type 2 *KLK8*, which functions as a splicing enhancer and creates a novel splicing site 8 bp upstream of the locus [19]. Type 2 *KLK8* is thus identical with type 1 except for a 135 bp (45 amino acid) insert located in exon 3 (Fig. 2a) [26]. Interestingly, human type 1 and type 2 *KLK8* mRNA transcripts produce the same active protease, as the type 2-specific 45-amino acid insertion is located outside the peptide sequence of the mature functional neuropsin protein [20].

**Fig. 3:**
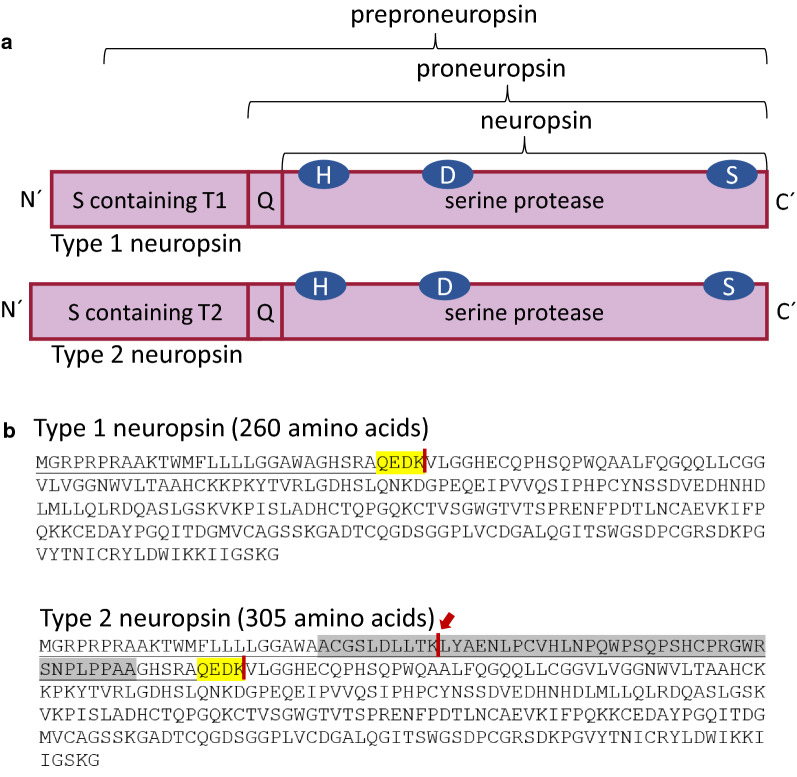
Human neuropsin preproprotein structure. **a** Shown are: the signal sequence (S) (containing either the type 1-specific amino acid sequence (T1) or type 2-specific amino acid sequence (T2)); the activity-masking peptide (Q) (QGSK in mice and QEDK in human); and the approximate amino acid locations of the characteristic catalytic triad of serine proteases (H, D, and S). **b** Human neuropsin preprotein amino acid sequence showing the signal sequence (underlined), the activity-masking peptide (yellow) and endoprotease splice sites (red line). Type 2 neuropsin shows a novel endoprotease site (red arrow) in the type 2-specific amino acid sequence (highlighted in grey)

*KLK8* translation results in a preproneuropsin containing a serine protease peptide, an activity-masking peptide, and a signal peptide [31] (Fig. 3a).

The signal peptide destines the preproneuropsin for translocation to the endoplasmic reticulum and eventually for secretion as a proneuropsin following its removal. Thus, a possible effect of the type 2-specific 45-amino acid insert might involve post-translational modifications (PTMs) or secretory properties. Applying the PTM prediction tool ModPred [32], the insert contained several likely modification sites (Additional file 1: Figure S1). Notably, the insert also resulted in an altered prediction profile of the downstream sequence mutual with type 1 neuropsin (Additional file 2: Table S2). It is thus conceivable, that protein folding or protein–protein interactions differ between type 1 and type 2 [20, 26]. On the other hand, supporting the notion of different secretory properties, cell-type specific secretion of neuropsin type 1 and 2 has been reported [20]. Proneuropsin is secreted via the regulated pathway in Neuro2a as well as PC12 cells [33]. Its secretion is stimulated by high K^+^ and has been shown to be calcium dependent [33]. The non-active proneuropsin is stored in the extracellular matrix (ECM), probably mostly in the synaptic cleft [34]. Activation of extracellular proneuropsin is facilitated by removal of the activity-masking peptide (QEDK) [35]. The responsible activating endoprotease has not yet been identified, but activation of proneuropsin follows various stimuli in mice, such as kindling epileptogenesis, long-term potentiation (LTP) and application of drugs that can depolarize synaptic activity [34]. Consequently, no or only a little protease activity has been detected in unstimulated brain tissues [2]. Wether neuronal stimuli also initiate secretion of the inactive preproneuropsin is, however, not yet clear. Interestingly, an extra endoprotease site has been identified in the type 2-specific 45-amino acid region (Fig. 3b) causing an intermediate protein form during the activation process [20]. Since protease activation of neuropsin only occurs after cleavage of the activity-masking peptide, this intermediate form shows very low amidolytic activity, like other proneuropsin forms [20].

## The neurobiology of neuropsin

Acting at the synapse, neuropsin has through its proteolytic activity been implicated with several molecular mechanisms underlying synaptic plasticity and associated cognitive and behavioral traits. The coding region of *KLK8* is relatively conserved between species [36], indicating that the molecular function of neuropsin has been evolutionary maintained. However, dynamic changes in *KLK8* TSS usage during primate evolution implies functional divergence of neuropsin in the CNS and species-specific roles of neuropsin in neurobiology. Upon its discovery, it was originally speculated that the human-specific type 2 *KLK8* may be important for adult brain plasticity, whereas *KLK8* in general may be necessary for the development of the human nervous system [26]. However, the detection of type 2 *KLK8* in the embryo brain [20] complicates such interpretation.

In mice, neuropsin has a regulatory effect on Schaffer-collateral LTP [37], which is important for the acquisition of hippocampus-associated memory. LTP describes a persistent strengthening of synapses based on recent patterns of activity and consists of two phases—the temporary early LTP (E-LTP) and the long-lasting late LTP (L-LTP). Whereas L-LTP requires protein kinase A (PKA) activation leading to altered gene expression and novel protein biosynthesis [38], E-LTP comprises an interaction between the ECM and synaptic membranes and thus mechanically modulates synaptic plasticity. It requires N-methyl-d-aspartate (NMDA) receptor and calcium/calmodulin-dependent protein kinase II (CAMKII) activation, which in turn increases the number of α-amino-3-hydroxy-5-methyl-4-isoxazolepropionic acid (AMPA) receptors at synapses [39]. Post-synaptic NMDA receptor activation following synaptic stimuli in E-LTP has been shown to induce a rapid activation of the precursor form of neuropsin [4].

**Fig. 4:**
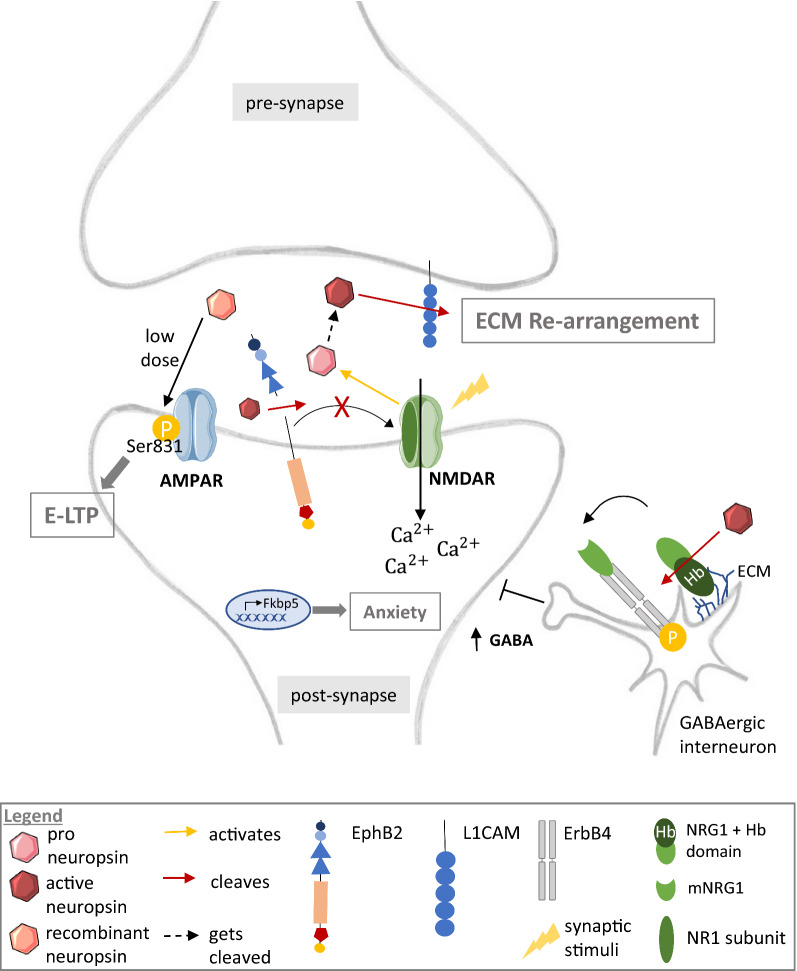
Neuropsin pathways implicated in synaptic plasticity. When an action potential arrives at the pre-synapse, this signal will be transmitted to the post-synapse by activating the NMDA receptor (NMDAR). NMDAR activation leads to the removal of the activity-masking peptide of proneuropsin (light red) and results in neuropsin activation. Furthermore, stress induces an increased *Klk8* expression. Active neuropsin (dark red) then cleaves (red arrow) its substrate L1CAM (blue), EphB2 (peach/blue) and NRG1 (green). L1CAM cleavage in the hippocampus might lead to an increased flexibility of synaptic structures. Extracellular cleavage of EphB2 in the amygdala prevents EphB2 and NMDAR clustering and hereby enhances the NMDAR current. This induces *Fkbp5* gene expression and behavioral signs of anxiety. After removal of the heparin binding domain (dark green, Hb) mature NRG1 (mNRG1, light green) binds to its receptor ErbB4 (grey) in GABAergic interneurons. Consequently, ErbB4 (grey) gets phosphorylated (yellow circle). ErbB4 phosphorylation is critical for GABAergic inhibitory transmission. Thus neuropsin-dependent NRG1 cleavage modulates E-LTP through the modulation of inhibitory projections into CA1 pyramidal cells in the hippocampus. Recombinant neuropsin furthermore leads to phosphorylation of the AMPA receptor (AMPAR) at a phosphorylation site specific for E-LTP (Ser831). Elements downloaded from [41]

Active neuropsin cleaves the extracellular domain of L1CAM and produces a neuropsin-specific 180-kDa fragment [4, 40] (depicted in Fig. 4).

The function of this neuropsin-specific L1CAM fragment has not been investigated yet, but the role of cell adhesion molecules in synaptic plasticity [42–45] suggests that neuropsin-specific L1CAM cleavage decreases synaptic adhesion and subsequently increases the flexibility of synaptic structures. Neuropsin is involved in the synaptogenesis of L1CAM expressing orphan and small synaptic boutons in pre-synaptic membranes of the Schaffer-collateral pathway in the hippocampal CA1 substructure [40]. In accordance, *Klk8*-deficient mice are characterized by abnormalities of synapses and neurons in the CA1 subfield of the hippocampus [46] and show significantly impaired E-LTP and memory acquisition, but not memory retention [47]. Similarly, in vivo inhibition of neuropsin in wild-type (WT) mice leads to impaired E-LTP [47], and delivery of recombinant neuropsin to the hippocampus, conversely, to increased synaptic transmission and AMPA phosphorylation at an E-LTP activating site [47] (depicted in Fig. 4). Collectively, this implies that neuropsin-dependent L1CAM processing might modulate activity-dependent structural changes involved in E-LTP. It has been suggested that neuropsin, based on its potential of extracellular modulation, might be important for the acquisition of memory depending on input strength [48]. A study in *Klk8*-deficient mice has demonstrated that neuropsin facilitates the association of two synapses in the apical and basal dendrites of CA1 pyramidal neurons, enabling a weak stimulus resulting in synaptic persistency, when being associated with a separate strongly stimulated pathway [48]. This phenomenon is referred to as synaptic tagging and facilitates the transformation of E-LTP to L-LTP without inducing novel protein biosynthesis.

Another pathway through which neuropsin may contribute to the modulation of synaptic plasticity involves NRG1, its receptor ErbB4 and GABAergic transmission in the CA1-3 subfields of the hippocampus [3] (depicted in Fig. 4). Activated by neuronal activity, neuropsin cleaves and releases NRG1 from its position in the ECM, which subsequently binds to its receptor ErbB4, leading to its phosphorylation [3]. ErbB4 is expressed in parvalbumin-positive GABAergic interneurons within the CA1 region and its phosphorylation is critical for GABAergic transmission [3]. Impairment of GABAergic transmission in *Klk8*-deficient mice leads to excessive post-synaptic excitation, which prevents the induction of NMDA receptor-dependent LTP [49]. Apart from that, the impairment of parvalbumin-expressing GABAergic interneuron activity contributes to cognitive disturbance which follows seizures as well as asynchronous network activity [50]. Parvalbumin-positive GABAergic interneurons coordinate the excitation/inhibition balance in the hippocampus by generating gamma oscillations [51, 52] and it has been found that neuropsin is involved in the generation of slow gamma oscillations via the NRG1–ErbB4 pathway [50]. Seizure activity induces neuropsin expression in the mouse brain [53] and has been shown to activate ErbB4-expressing parvalbumin-positive interneurons via neuropsin–NRG1 signaling [50]. Consistently hippocampal gamma oscillations are impaired in neuropsin-KO mice [50].

Linking the function of neuropsin to behavioral regulation, mice exposed to acute or chronic stress show increased *Klk8* mRNA expression in hippocampal tissue accompanied by depressive-like behavior [15]. This upregulation of *Klk8* after stress exposure was shown to be dependent on the stress hormone corticosterone in primary cultured hippocampal neurons as well as in vivo [14, 15]. Corticosterone exposure causes impairment in spatial memory, neurogenesis, dendritic morphology and demyelination in WT mice, while mice lacking *Klk8* (knockout mice as well as knockdown of *Klk8* by viral vectors) appear protected against these effects as well as the development of depressive-like behavior [15]. In line with this, overexpression of *Klk8* by viral vectors led to an increased impairment in spatial memory and depressive-like behavior. Neuropsin has further been demonstrated to play a critical role in stress-related plasticity in the amygdala, where stress-induced neuropsin-dependent EphB2 cleavage leads to the dissociation of EphB2 from the NR1-subunit of the NMDAR [5]. This changes the dynamics of the EphB2–NMDA-receptor interaction in mice, enhances NMDAR current and induces anxiety-like behavior [5]. *Klk8*-deficient mice, however, are protected against stress-induced EphB2 cleavage and the associated behavioral changes [5].

## *Klk8* in mental disorders

Despite its documented role in synaptic signaling and importance in neurobiology and behavior, no clinical data directly link neuropsin to any brain disorder, including mental illnesses. However, there are genetic and epigenetic findings that support its implication in human mental health. This includes clinical data from individuals carrying structural genetic variants encompassing *KLK8*, among which > 60% present with intellectual disability [54]. Other associated mental health phenotypes include specific learning disabilities, seizures and autism [54]. However, *KLK8* is one of 15 kallikrein subfamily members located in tandem in a gene cluster on chromosome 19, so phenotypes cannot be specifically attributed to changes in *KLK8* copy number. Although a rare (< 0.001% [55]) missense Val286Ile mutation has been identified in a schizophrenia (SZ) case [56] (Table 1), no other disruptive rare *KLK8* variants have been reported in any of the major exome or copy number variation studies performed on SZ or autism spectrum disorder (ASD) cases [57]. Thus, supporting the finding that *KLK8* is tolerant to loss of function (LOF) variants [54, 55].

**Table 1:** Descriptive overview of human studies, in which *KLK8* was associated to mental health phenotypes

Phenotype	Tissue	Parameters assessed	Main finding	Reference
Schizophrenia	Peripheral blood	Singe nucleotide polymorphisms (genotyping assay)	Rare Val286Ile missense mutation in exon 6 detected in a patient with schizophrenia	[56]
Bipolar disorder	Peripheral blood	Singe nucleotide polymorphisms (genotyping assay)	Significant allelic association between several SNPs and bipolar disorder: rs1722550 (P = 0.019), rs1701946 (P = 0.018), rs1612902 (P = 0.002), rs1701946 plus rs1612902 (P = 0.0068)	[56]
Depression	Peripheral blood	Expression levels (mRNA) of *KLK8* (RT-PCR), patients diagnosed with major recurrent depression and first episode depression	Higher *KLK8* expression in patients with recurrent depression compared to first episode patients	[64]
	Peripheral blood	Expression levels (mRNA) of *KLK8* (RT-PCR), patients diagnosed with major recurrent depression and healthy subjects	Higher *KLK8* expression in patients with depression compared to controls	[63]
	Peripheral blood	Methylation levels of *KLK8* (450 K methylation array*),* depression symptomatology score in monozygotic twins	Methylation levels in the promotor region of *KLK8* is associated with depression symptomatology in general population	[65]
Alzheimer’s disease	Hippocampal and parietal cortex	Expression (RT-PCR), of *KLK8* in Alzheimer’s disease (AD) and control tissue	11.5-fold increase in *KLK8* mRNA levels in AD hippocampus compared to controls	[66]

**Fig. 5:**
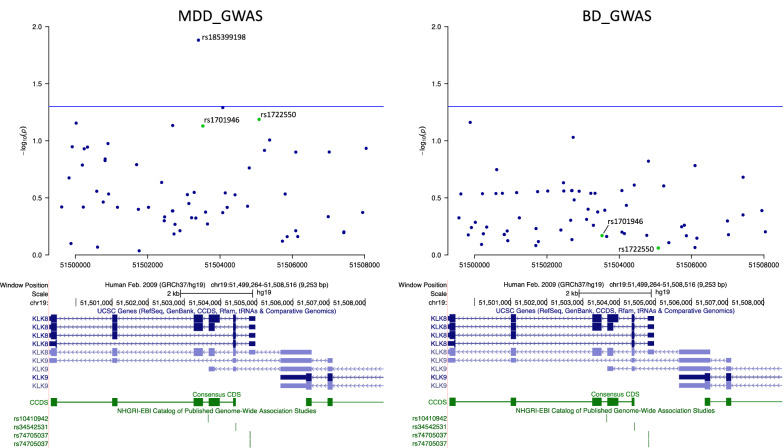
Single nucleotide polymorphisms (SNPs) associated with mental disorders. Shown are SNPs identified in MDD and BD GWASs [58, 61] as well as two SNPs (rs rs1722550 and rs1701946) associated with bipolar disorder [56] in relation to the UCSC genomic sequences of *KLK8* splice variants in blue. Blue line: nominal significant threshold of *p* < 0.05. Asterisk indicate SNP located 3′ to *KLK8*

A candidate study assessing genetic association between *KLK8* polymorphisms and BD and SZ, identified an association between BD and three SNPs, as well as a two-marker haplotype (Table 1) [56]. Interestingly, healthy individuals carrying the BD risk allele of *KLK8*, rs1612902, showed a lower score in attention/concentration and verbal IQ [56]. However, in terms of common variation, no genome-wide significant association could be found between variants in the *KLK8* locus and mental disorders in the currently largest major depressive disorder (MDD), SZ, bipolar disorder (BD), ASD or attention deficit hyperactivity disorder (ADHD) genome-wide association studies (GWASs) [58–62] (Fig. 5 and Additional file 1: Figure S2).

Assessment of *KLK8* mRNA levels in peripheral blood from 186 patients diagnosed with major recurrent depression (MRD) compared to 105 healthy subjects, revealed significantly higher *KLK8* expression in patients [63]. Furthermore, it was shown that *KLK8* mRNA is significantly more abundant in blood samples from patients affected by MRD, compared to patients suffering from first episode depression. The observed increase in *KLK8* expression was associated with diminished interpersonal abilities in depressive patients [64]. An association between depression symptomatology score in the general population and blood DNA methylation levels in the promoter region of *KLK8* was recently identified in a large cohort of monozygotic Danish twins [65], supporting the implication of *KLK8* in depression symptomatology. However, a screening of *KLK8* expression data from RNA sequencing expression experiments on post-mortem brain tissues from MDD, BD and SZ patients, did not reveal significant differences between patients and healthy controls in any of the identified studies (Additional file 1: Figures S3 and S4). *KLK8* expression levels have, on the other hand, been reported to be 11.5-fold increased in the hippocampus of patients suffering from the neurodegenerative mental disorder Alzheimer´s disease (AD) compared to controls [66]. In line with this, RNA sequencing in post-mortem lateral temporal lobe tissue revealed a tendency of higher *KLK8* expression in aged AD patients compared to age-matched healthy individuals (GSE104704, Additional file 1: Figure S4).

## Limitations

In this review, we have only assessed available RNA sequencing data, while leaving out data generated from microarray-based platforms. Limited coverage and design of RNA sequencing experiments preclude transcript-specific analyses.

## Conclusion

The proteolytic specificity of neuropsin directed at synaptic proteins implicated in mental disorders, makes it an interesting candidate in molecular psychiatry. Brain-specific splicing of *KLK8* mRNA is only reported in humans and its expression and activation characteristics links it to neuronal activity and a neuro-molecular response to stress. Under non-pathological conditions, *KLK8* expression in the CNS is, however, weak and appears to be restricted to only a small subset of cells. Through systematic interrogation of available large-scale genetic and post-mortem brain transcriptomic studies, we do not find compelling clinical data that support *KLK8* dysregulation in mental illness. However, the endoprotease and molecular machinery responsible for neuropsin activation remains to be characterized and it is possible that post-translational regulation of neuropsin may be the dominating molecular mechanism of activity regulation in the mature brain.

Interestingly, *KLK8* expression measured in blood as well as cerebrospinal fluid is a promising early biomarker for AD as well as mild cognitive impairment due to AD [67]. Blood *KLK8* levels have, furthermore, successfully been established as a biomarker for cancer diagnosis [68]. It is, thus, possible that *KLK8* blood parameters levels may accordingly serve as diagnostic biomarkers in mental disorders. This is especially interesting when considering that both blood *KLK8* mRNA and methylation status have been associated with depression symptomatology [63, 65].

### Supplementary information

**Additional file 1: Table S1.***KLK8* expression in human brain single cells. Summarized are *KLK8* expression data from several studies [1–4] and databases [5–7]. **Figure S1.** Amino acid sequence of type 1 (top panel) and type 2 (bottom panel). **Figure S2.** Single nucleotide polymorphisms (SNPs) associated with mental disorders. **Figure S3.** Flowchart describing the dataset search in MEDLINE and GEO. **Figure S4***KLK8* expression in mental disorders.

**Additional file 2: Table S2.** Output from ModPred. Positions marked in green are positions predicted to feature PTMs.

## Data Availability

Data are freely available from public repositories with references hereto included in the main text.

## Abbreviations

AD: Alzheimer’s disease
AMPA: α-Amino-3-hydroxy-5-methyl-4-isoxazolepropionic acid
BD: Bipolar disorder
CAMKII: Calcium/calmodulin-dependent protein kinase II
ECM: Extracellular matrix
E-LTP: Early long-term potentiation
EphB2: Eph receptor B2
GWAS: Genome-wide association study
KLK8: Neuropsin gene
LOF: Loss of function
LTP: Long-term potentiation
L-LTP: Late long-term potentiation
L1CAM: Cell adhesion molecule L1
MDD: Major depressive disorder
MRD: Major recurrent disorder
NMDA: N-Methyl-d-aspartate
NRG-1: Neuregulin-1
PKA: Protein kinase A
SNP: Single nucleotide polymorphism
SZ: Schizophrenia
WT: Wildtype
