# Supplementary material for: Neuropsin in mental health
Source: J Physiol Sci. 2020 May 15;70:26. doi: 10.1186/s12576-020-00753-2 (PMC10717651; doi:10.1186/s12576-020-00753-2)
Supplement: Supplementary file 1 — Additional file 1: Table S1.KLK8 expression in human brain single cells. Summarized are KLK8 expression data from several studies [1–4] and databases [5–7]. Figure S1. Amino acid sequence of type 1 (top panel) and type 2 (bottom panel). Figure S2. Single nucleotide polymorphisms (SNPs) associated with mental disorders. Figure S3. Flowchart describing the dataset search in MEDLINE and GEO. Figure S4KLK8 expression in mental disorders. [file 12576_2020_753_MOESM1_ESM.pdf]

## Additional Figures and Tables

**Additional file1: Table S1:** *KLK8* expression in human brain single cells. Summarized are *KLK8* expression data from several studies [1-4] and databases [5-7]. Shown are fractions of cells with *KLK8* expression counts (in ‰) in neuronal as well as non-neuronal cells in several tissues and developmental stages.

| | Tissue | Developmental stage of donors | Number of nuclei | Fraction of cells with *KLK8* counts (‰) | | | |
|---|---|---|---|---|---|---|---|
| | | | | Neuronal | | Non-neuronal | Unclassified |
| | | | | Excitatory | Inhibitory | | |
| **Allen Brain Atlas** | Primary visual cortex | Adult | 8998 | 4 /8063 (0.50) | | 0/935 | - |
| | Anterior Cingulate Cortex | Adult | 7283 | - | - | - | - |
| | Middle temporal gyros | Adult | 15928 | 37/10525 (3.52) | 8/4164 (1.92) | 1/914 (1.09) | - |
| **GTEX** | Prefrontal cortex | Adult | 5932 | - | - | - | - |
| | Hippocampus | Adult | 9036 | 0/3501 | 0/1061 | 0/3773 | 6/701 (8.56) |
| **Linnarsson et al.** | Ventral midbrain | Embryo | 1977 | - | - | - | - |
| | Middle temporal gyrus cortex | Adult | 2028 | - | - | - | - |
| **Zhong et al.** | Prefrontal cortex | Embryo | 2309 | - | - | - | - |
| **Darmanis et al.** | Temporal lobe | Adult and fetal | 466 | 5/122 (40.98) | | 1/206 (4.85) | 0/138 |
| **PsychEncode** | Neocortex | Fetal | 115 | 1/40 (25) | 0/28 | 0/47 | - |
| | Frontal cortex | Fetal | 249 | 0/69 | 2/38 (52.63) | 1/142 (7.04) | - |
| | Pallium | Fetal | 398 | 0/0 | 2/143 (13.98) | 2/255 (7.84) | - |
| | Frontal cortex | Adult | 17093 | 0/8957 | 0/3721 | 0/4415 | - |

**Additional file2: Table S2 (see attached excel sheet):** Output from ModPred. Positions marked in green are positions predicted to feature PTMs. Positions marked in blue are type-specific predictions downstream of the 45 amino acid insert of type-2 *KLK8.* Asterix indicates position with non-shared PTM predictions.

**Additional file1: Figure S1:** Amino acid sequence of type 1 (top panel) and type 2 (bottom panel). *KLK8* with positions containing predicted PTMs color coded by level of confidence. Red rectangular marks the type 2 specific sequence.

***KLK8* type 1** (KLK8-211 peptide)

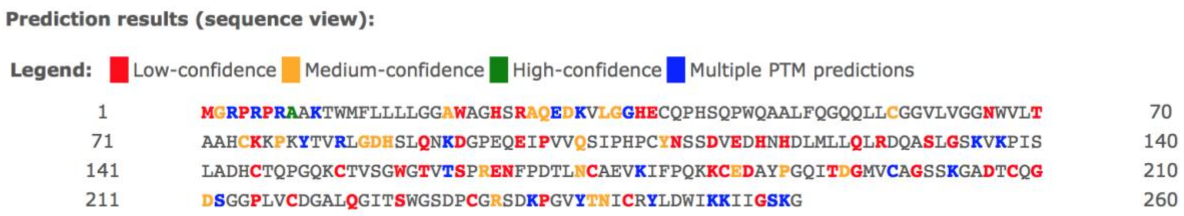


***KLK8* type 2** (KLK8-204 peptide)

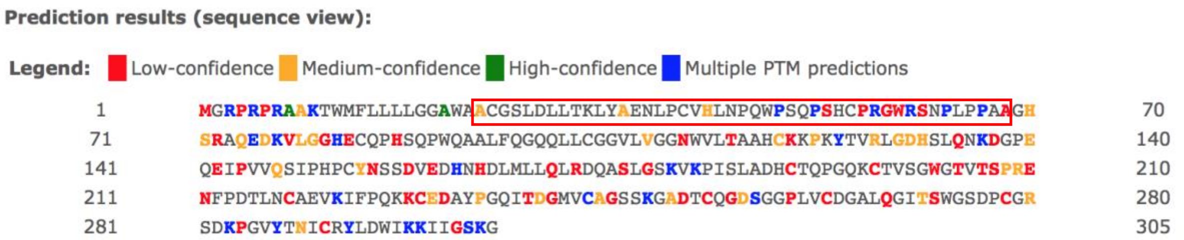

**Additional file1 : Figure S2.** Single nucleotide polymorphisms (SNPs) associated with mental disorders. **A)** In the currently largest genome-wide association studies (GWASs) no significant association has been found between variants in the *KLK8* locus and the mental disorders major Schizophrenia (SZ), autism spectrum disorder (ASD) or attention deficit hyperactivity disorder (ADHD) [8-10]. However, three SNPs identified in a genotyping assay [11] show an association with BD and are depicted here in the context of GWAS data. **B)** Shown are SNPs identified in GWASs in relation to the UCSC genomic sequences of *KLK8* splice variants in blue. Blue line: nominal significant threshold of $p < 0.05$. Asterix indicate SNP located 3' to *KLK8.*

**A**

| | MDD PGC + UKB | | BD PGC | | SZ PGC | | ASD iPSYCH | | ADHD iPSYCH | |
|---|---|---|---|---|---|---|---|---|---|---|
| SNP | OR | P | OR | P | OR | P | OR | P | OR | P |
| rs1722550 | 0.985 | 0.065 | 0.998 | 0.868 | 1.005 | 0.653 | 0.984 | 0.343 | 0.988 | 0.449 |
| rs1701946 | 0.986 | 0.074 | 1.009 | 0.498 | 1.002 | 0.889 | 0.977 | 0.163 | 0.986 | 0.391 |
| rs1612902* | 0.986 | 0.086 | 1.006 | 0.678 | 1.003 | 0.807 | 0.984 | 0.306 | 0.987 | 0.407 |

**B**

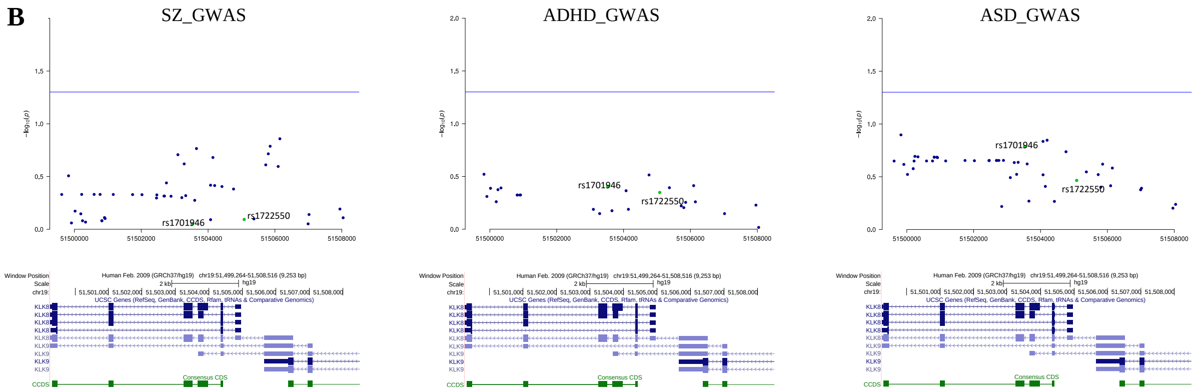

**Additional file1: Figure S3.** Flowchart describing the dataset search in MEDLINE and GEO. The last three steps in the flowchart describe the downstream workflow in R for identifying studies reporting *KLK8* expression levels in mental disorders.

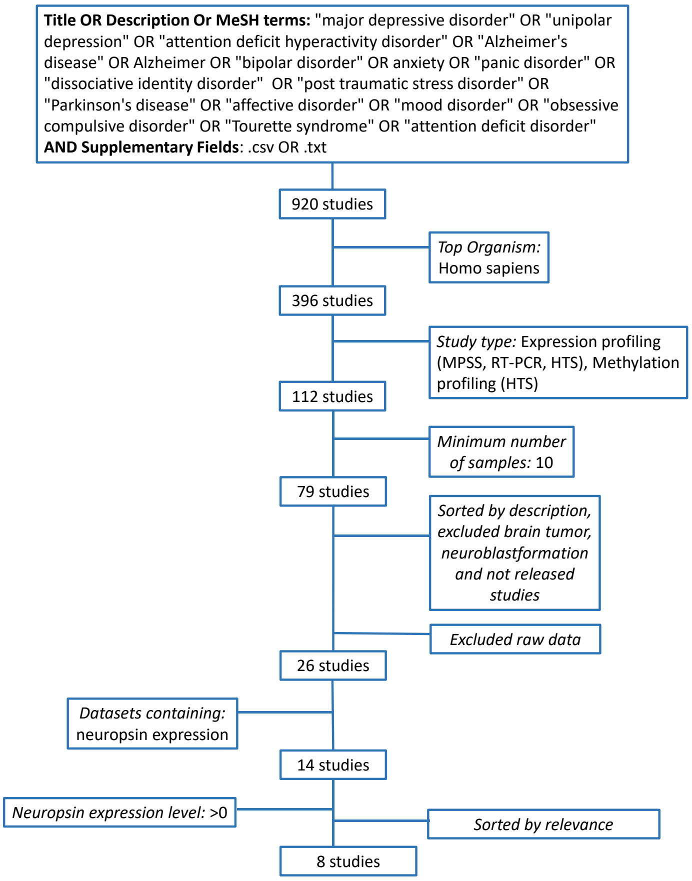

**Additional file1: Figure S4.** *KLK8* expression in mental disorders. Shown are *KLK8* expression levels by age in several brain tissues and several mental disorder phenotypes as well as healthy controls from selected studies identified as described in Supplementary Figure A.3. In GSE80655 the transcriptome in 281 clinically annotated human post-mortem brain tissues has been measured. GSE104704 compared the genome-wide enrichment of H4K16ac in the lateral temporal lobe of AD individuals against both younger and elderly cognitively normal controls. GSE101521 conducted whole-transcriptome brain expression profiling in MDD and suicide. GSE112523 studied DNA methylation in neurons from post-mortem brains in SZ and BD and GSE102556 combined differential expression and gene co-expression network analyses to provide a comprehensive characterization of male and female transcriptional profiles associated with MDD across six brain regions. Screening of the data from those studies did not reveal significant differences in *KLK8* expression levels between MDD, BD or SZ patients and healthy controls.

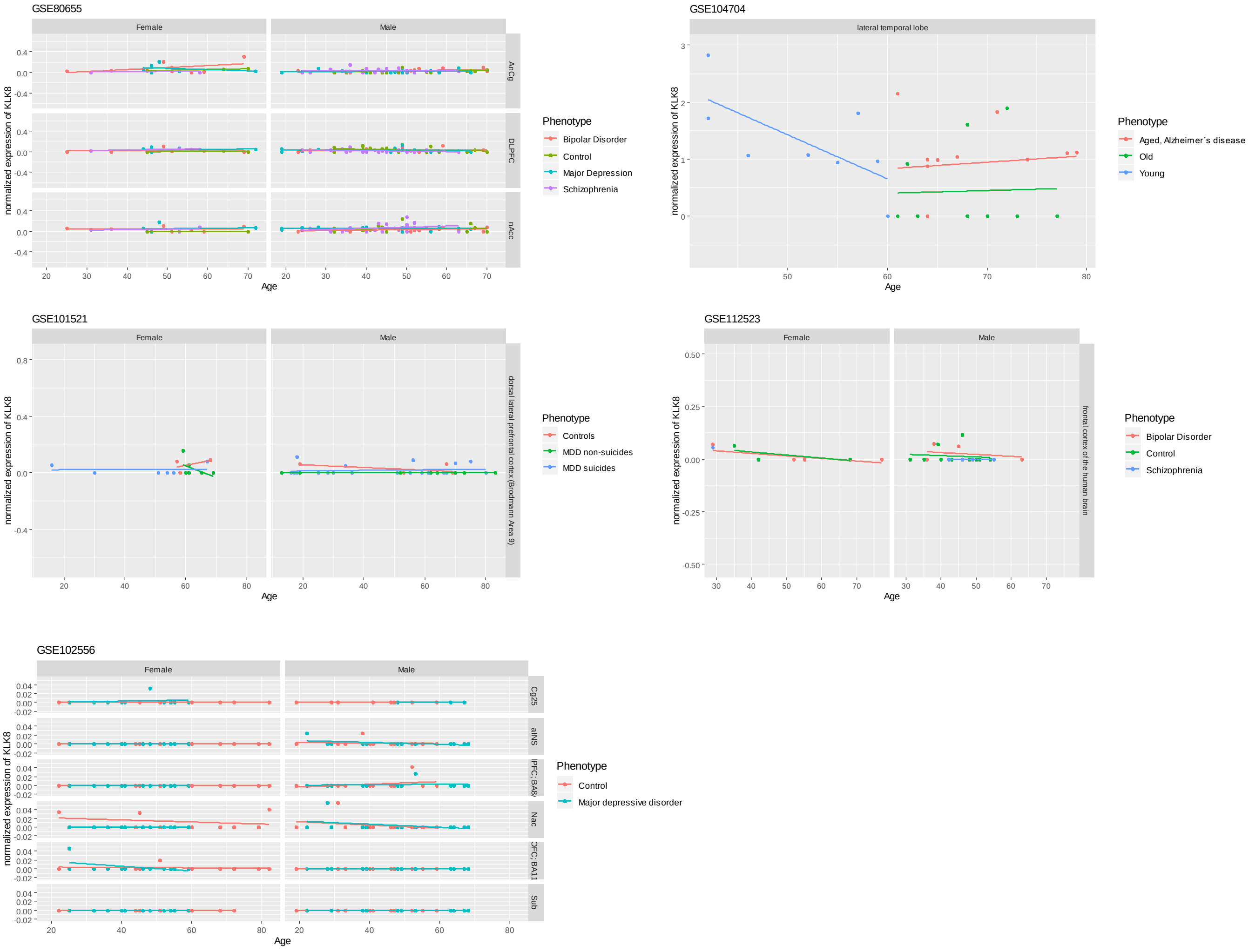

References:


1. La Manno, G., et al., *Molecular Diversity of Midbrain Development in Mouse, Human, and Stem Cells.* Cell, 2016. **167**(2): p. 566-580 e19.
2. Zeisel, A., et al., *Brain structure. Cell types in the mouse cortex and hippocampus revealed by single-cell RNA-seq.* Science, 2015. **347**(6226): p. 1138-42.
3. Zhong, S., et al., *A single-cell RNA-seq survey of the developmental landscape of the human prefrontal cortex.* Nature, 2018. **555**(7697): p. 524-528.
4. Darmanis, S., et al., *A survey of human brain transcriptome diversity at the single cell level.* Proc Natl Acad Sci U S A, 2015. **112**(23): p. 7285-90.
5. Habib, N., et al., *Massively parallel single-nucleus RNA-seq with DroNc-seq.* Nat Methods, 2017. **14**(10): p. 955-958.
6. Wang, D., et al., *Comprehensive functional genomic resource and integrative model for the human brain.* Science, 2018. **362**(6420).
7. Science, A.I.f.B. *Allen Human Brain Atlas*. 2010; Available from: https://portal.brain-map.org/atlases-and-data/rnaseq.
8. Schizophrenia Working Group of the Psychiatric Genomics, C., *Biological insights from 108 schizophrenia-associated genetic loci.* Nature, 2014. **511**(7510): p. 421-7.
9. Grove, J., et al., *Identification of common genetic risk variants for autism spectrum disorder.* Nat Genet, 2019. **51**(3): p. 431-444.
10. Demontis, D., et al., *Discovery of the first genome-wide significant risk loci for attention deficit/hyperactivity disorder.* Nat Genet, 2019. **51**(1): p. 63-75.
11. Izumi, A., et al., *Genetic variations of human neuropsin gene and psychiatric disorders: polymorphism screening and possible association with bipolar disorder and cognitive functions.* Neuropsychopharmacology, 2008. **33**(13): p. 3237-45.
